# Insulin-Enhanced Biological Visual Rehabilitation in Neuroretinal Degeneration Patients Treated with Mesenchymal Cell-Derived Secretome

**DOI:** 10.3390/pharmaceutics17070901

**Published:** 2025-07-11

**Authors:** Paolo Giuseppe Limoli, Celeste Limoli, Marcella Nebbioso

**Affiliations:** 1 Low Vision Research Centre of Milan, p.zza Sempione 3, 20145 Milan, Italy; paololimoli@libero.it (P.G.L.); celeste.limoli@unimi.it (C.L.); 2 Department of Sense Organs, Faculty of Medicine and Odontology, Sapienza University of Rome, P. le A. Moro 5, 00185 Rome, Italy

**Keywords:** insulin-like growth factor (IGF), Limoli retinal restoration technique (LRRT), insulin lispro, microperimetry, neurodegenerative retinal diseases, topical insulin

## Abstract

**Objectives:** Insulin plays a crucial role in neuronal survival and oxidative stress modulation, making it a potential therapeutic target. This study investigates the effects of insulin in combination with a mesenchymal cell-derived secretome in patients with degenerative neuroretinal diseases. **Methods:** Sixty-four patients with severe neuroretinal diseases who had previously undergone the Limoli Retinal Restoration Technique (LRRT) were included in this longitudinal study and divided into groups: group 1 received a single injection of 5 units of insulin lispro into the suprachoroidal space of the worse-seeing eye; group 2 received insulin injection in the better-seeing eye. Retinal function was assessed using microperimetry (MY) before and after treatment (approximately 1 year for eye drops). Group 3 consisted of patients who demonstrated improvement in MY after insulin injection. These patients continued treatment with daily insulin eye drops. **Results:** In group 1, insulin-treated eyes showed a significant increase in retinal sensitivity from 10.09 dB to 10.75 dB (*p* = 0.0067), while untreated eyes declined from 12.35 dB to 11.92 dB (*p* = 0.0448). In group 2, insulin-treated eyes improved from 10.8 dB to 11.63 dB (*p* = 0.05), whereas untreated eyes exhibited a decline from 8.68 dB to 8.50 dB (*p* = 0.6771). In group 3, patients using insulin eye drops showed a stabilization or mild increase in retinal sensitivity, from 11.39 dB to 11.73 dB (*p* = 0.231). **Conclusions:** The addition of insulin in patients previously treated with the LRRT was associated with improved sensitivity and a stabilizing effect on neuroretinal function.

## 1. Introduction

The physiological function of the eye depends on the metabolic homeostasis of neuroretinal cells, which is essential for maintaining cellular integrity and supporting normal visual processing. The vulnerability of these cells to metabolic stress is evident in conditions such as severe hypoglycemia, where a rapid decline in glucose availability leads to functional deficits and visual impairment [1].

**Figure 1:**
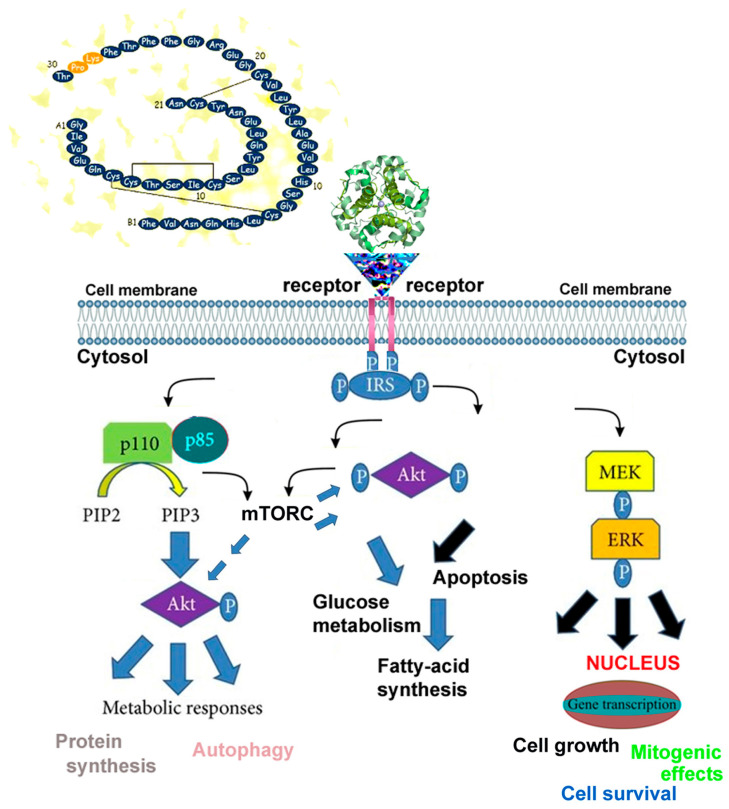
Simplified schematic representation of insulin-mediated oxidative phosphorylation and neuroretinal cell viability. Insulin or insulin-like growth factor 1 (IGF-1) binds to its respective receptor, activating receptor tyrosine kinase activity. This activation phosphorylates intracellular substrates, initiating complex signaling cascades involving molecules such as p110, p85, PIP2, PIP3, MEK, and ERK (yellow, blue, and black arrows). These pathways regulate critical metabolic processes, including glycolysis and oxidative phosphorylation, which produce adenosine triphosphate (ATP) essential for nerve signal transmission and ion gradient maintenance in neuroretinal cells. AKT (also known as protein kinase B, PKB) acts as a central node, activated downstream to promote protein synthesis via mTORC1-a serine/threonine kinase complex that senses nutrient, energy, and stress levels and regulates cell growth, proliferation, and metabolism.

Chronic metabolic dysregulation and impaired cellular energy homeostasis have also been implicated in the pathogenesis of retinal degenerative diseases, including diabetic retinopathy (DRP), age-related macular degeneration (AMD), and inherited retinal dystrophies [2,3,4]. Given the fundamental role of metabolism in neuroretinal health, targeting metabolic pathways represents a promising therapeutic strategy for modulating disease progression and preserving visual function [2,3,4]. Insulin, a key regulator of cellular metabolism, facilitates glucose uptake in neuroretinal cells, including neurons, Müller cells, and retinal ganglion cells (RGCs), by stimulating transporters such as GLUT1 and GLUT3. It also activates critical metabolic pathways, such as the PI3K/AKT pathway, which promotes neuronal survival, and it regulates glycolysis and oxidative phosphorylation, thereby supporting adenosine triphosphate (ATP) production essential for cellular processes such as synaptic activity and ion transport. In neurodegenerative retinal diseases such as PKB (GL) and AMD, impaired metabolism can lead to oxidative stress, mitochondrial dysfunction, and deficits in neuronal excitability. These disturbances contribute to neuroretinal damage, in which insulin signaling may play a protective role by promoting metabolic stability and enhancing cell survival. Therefore, under pathological conditions, metabolic impairment in the neuroretina may contribute to cellular dysfunction and eventual apoptosis. Insulin signaling may help mitigate these disturbances, potentially supporting both cell survival and function [5,6] (Figure 1).

The mesenchymal cell-mediated secretome delivered via the Limoli Retinal Restoration Technique (LRRT) represents an emerging approach to retinal neuroprotection. This technique involves the implantation of mesenchymal cells—including adipose stromal cells (ASCs) derived from orbital fat, adipose-derived stem cells (ADSCs) obtained from abdominal fat through liposuction and centrifugation, and platelet rich plasma (PRP) into the suprachoroidal space [7]. The secretome produced by these cellular components exerts trophic, anti-inflammatory, anti-apoptotic, and hemorheological actions within the retinal microenvironment, with the aim of stabilizing or slowing the progression of neuroretinal degenerative diseases [8,9,10,11].

These cells release a variety of bioactive molecules, including insulin-like growth factors (IGFs), which share structural and functional similarities with insulin in supporting cellular metabolism and survival (Figure 1). The secretome also includes growth factors (GFs), chemokines, and cytokines, present both as soluble proteins and within extracellular vesicles that carry mRNA, ribosomes, and mitochondria, all of which may influence cellular function [7,8,9,10,11]. Emerging evidence suggests that the mesenchymal secretome may help slow the progression of neuroretinal diseases by modulating neuroretinal cells through paracrine signaling, thereby promoting cellular stability and function [12,13,14,15,16,17,18]. IGFs have also been increasingly recognized as a potential therapeutic target in a variety of conditions, including osteoporosis, diabetes, obesity, neuromuscular disorders, and resistance to growth hormone and insulin [19,20,21]. The improved cellular metabolism resulting from interaction with the mesenchymal secretome—particularly with factors such as IGF—may underlie the functional improvements observed following suprachoroidal mesenchymal implantation in retinal neurodegenerative diseases [22,23,24,25]. Despite its potential benefits, the combined effects of insulin and the mesenchymal secretome have yet to be fully elucidated.

Insulin lispro is a rapid-acting analog of human insulin, commonly used to treat type 1 and type 2 diabetes via subcutaneous administration. Its onset of action occurs within approximately 30 min, with effects lasting about 5 h. It functions similarly to endogenous insulin by increasing peripheral glucose uptake and decreasing hepatic glucose production [26].

Insulin lispro is a manufactured analog of human insulin in which the amino acids lysine and proline have been swapped at the C-terminal end of the B-chain. Using recombinant DNA technology, the final lysine and proline residues at positions 28 and 29 on the B-chain are reversed. This modification mimics IGF-1, which also contains lysine and proline at these positions [27,28].

The alteration does not affect receptor binding but prevents the formation of insulin dimers and hexamers, allowing larger amounts of active monomeric insulin to be immediately available following postprandial injections [29].

Common side effects include skin irritation at the injection site, hypoglycemia, hypokalemia, lipodystrophy, anaphylaxis, and hypersensitivity reactions. Insulin lispro was first approved for use in the United States in 1996 and received marketing authorization in April 1996 by the European Medicines Evaluation Agency (EMEA) for European Union Countries and by the Food and Drug Administration (FDA) [28,29].

Few studies have investigated the potential interactions between insulin and mesenchymal-derived bioactive molecules in modulating cellular function and tissue repair, particularly in the context of neurodegenerative retinal diseases [30,31,32]. It remains unclear whether insulin can enhance the effects of growth factors such as IGF due to overlapping signaling pathways. Thus, the specific role of insulin in modulating neuroretinal cell function under pathological conditions remains unclear [33].

The aim of this study is to evaluate whether insulin, administered in addition to mesenchymal cell-derived secretome treatment implanted at the suprachoroidal level, can significantly influence neuroretinal cell function as measured by retinal microperimetry (MY) in patients with various neurodegenerative retinal diseases.

## 2. Materials and Methods

This longitudinal study included 64 patients with severe neuroretinal diseases who attended the Low Vision Center in Milan between January 2020 and September 2024, allowing for reliable longitudinal assessment.

All procedures performed in studies involving human participants were in accordance with the ethical standards of the Institutional and National Research Committee and with the 1964 Helsinki Declaration and its later amendments or comparable ethical standards. The Institutional Review Board (IRB) of the Low Vision Academy reviewed and approved the study protocol (No. 2019/A928, dated 28 September 2019), which waived the requirement for formal ethical approval due to the nature of the study. All participants provided written informed consent to participate in the research. We a priori-selected a cohort of patients with various neuroretinal diseases, as these conditions exhibit a progressive nature that allows for a broader assessment of therapeutic interventions across different pathologies. Severe neuroretinal diseases typically have an insidious onset and progressively worsen over time, leading to damage of the retina and optic nerve, and ultimately resulting in vision loss. Their development may be influenced by multiple factors, including genetic predisposition, immune dysregulation, vascular dysfunction, aging, and chronic inflammation. Due to their heterogeneous nature and progressive course, no single parameter can fully capture the extent of dysfunction. Visual field loss may involve central or peripheral regions or be confined to specific localized areas.

Based on these considerations, the inclusion criteria for the study were defined as follows: patients diagnosed with glaucomatous optic neuropathy (GON), angioid streaks (AS), myopic macular degeneration (MMD), MMD combined with GON, rod–cone dystrophy (RCD), Stargardt’s macular degeneration (SMD), optic atrophy (OA), retinitis pigmentosa (RP), diabetic retinopathy (DRP), dry age-related macular degeneration (AMD), occult macular dystrophy (OMD), cone dystrophy (CD), or gyrate atrophy of the choroid (GAC).

These patients, aged between 23 and 88 years, had previously undergone the LRRT, a suprachoroidal autologous mesenchymal cell implantation aimed at stabilizing or slowing the progression of their respective diseases. To maintain the therapeutic effect, patients received periodic suprachoroidal injections of 1.5 mL platelet-derived mesenchymal secretome into the area previously implanted with LRRT. The secretome, prepared by centrifugation and calcium gluconate-induced α-granule degranulation, was administered under sterile surgical conditions [34,35].

All patients had undergone LRRT months to years prior to the study. To preserve the trophic activity of the implanted peduncle and its anti-apoptotic effects on the neuroretina, suprachoroidal injections of the secretome were repeated every 6–12 months. The last injection before the addition of insulin was administered no more than 6–12 months earlier.

Moreover, several studies have demonstrated that the activity of the secretome can be modulated by altering the cellular microenvironment or by supplementing it with specific bioactive molecules [36,37].

Additionally, best-corrected visual acuity (BCVA) inclusion criteria required a range between 2 and 0 logarithm of the minimum angle of resolution (logMAR) for distance vision and between 12 and 6 points for near vision.

The exclusion criteria included subjects treated for ocular or systemic pathologies, such as uveitis, cataract, retinal detachment, ischemic or inflammatory neuropathy, ocular vascular occlusion, strabismus, and ocular trauma, or suffering from systemic conditions, including cancer, stroke, previous severe systemic trauma, uncontrolled diabetes mellitus, hypertension, and/or uncompensated heart disease, among others.

All patients received an injection of 1.5 cc of mesenchymal secretome—obtained through platelet centrifugation and alpha-vesicle degranulation using calcium gluconate—into the suprachoroidal peduncle at the site of the previously implanted LRRT. The secretome was further supplemented with 5 units (Us) of insulin lispro administered via Humalog 100 U/mL KwikPen (Humalog, Eli Lilly, Sesto Fiorentino, Florence, Italy). Blood glucose levels were monitored for the first 6 h post injection. To prevent hypoglycemic events potentially caused by systemic insulin diffusion, patients received a slow intravenous infusion of 100 mL of 5% glucose solution following secretome and insulin injection. The therapeutic effect of insulin combined with the mesenchymal secretome via the LRRT was assessed by measuring retinal function and sensitivity using MY (MAIA, CenterVue S.p.A., Padova, Italy). Measurements were taken at 180 and 60 days before treatment and at 3 and 90 days after treatment.

To evaluate treatment efficacy both subjectively and objectively, a standardized scoring system was employed to quantify patient-reported changes and clinical observations. Patients were assigned a subjective score based on perceived improvement (+1), stability (0), or worsening (−1). Similarly, an objective score was assigned based on clinical judgment: improvement (+1), stability (0), or worsening (−1).

Patients were divided into three groups. In the first group, consisting of 39 patients (78 eyes), the worse-seeing eye received an injection of 5 units of insulin into the previously implanted suprachoroidal peduncle, while the better-seeing eye received no insulin. In the second group, comprising 25 patients (50 eyes), the better-seeing eye received a suprachoroidal injection of 5 units of insulin into the previously implanted peduncle, while the worse-seeing eye remained untreated. The better eye was treated at a later stage. A third group of 26 patients (45 eyes) was made up of subjects from the first and second groups who demonstrated improved retinal sensitivity following insulin peduncular injection. Patients continued therapy with daily eye drops composed of 80% insulin lispro (Humalog, Eli Lilly, 100 U/mL, Sesto Fiorentino, Florence, Italy;) and 20% hyaluronic acid (2%). The eye drops were administered once daily in the morning, following breakfast, to minimize the risk of hypoglycemia associated with administration on an empty stomach.

Blood glucose levels were measured one hour after eye drop administration and compared to baseline values obtained one hour after breakfast, prior to initiating treatment. Patients continued daily use of the eye drops for approximately one year.

Retinal sensitivity was assessed at the beginning of treatment and again after a mean duration of 346.64 days (range: 150–728 days). Blood glucose was monitored at home using Dextrostix (Bayer, Leverkusen, Germany), one hour after breakfast, both before starting the therapy and on the first day of treatment.

Daily use of the insulin eye drops was not associated with any significant local or systemic adverse events. Locally, some patients reported a transient burning sensation upon instillation. Systemically, when the drops were used without prior food intake, a few patients experienced brief episodes of weakness and hypotension, which resolved promptly after consuming carbohydrates such as bread, a croissant, or sweetened coffee.

Statistical analysis. Continuous variables are expressed as means ± standard deviations (SDs), while categorical variables are presented as frequencies and percentages. Within-group comparisons were performed using paired *t*-tests. Categorical variables were compared using Fisher’s exact test. Data distribution was assessed for normality with the Shapiro–Wilk test, which confirmed a normal distribution. Statistical significance was defined as *p* < 0.05. All analyses were conducted using SPSS Statistics version 29.0 (SPSS Inc., Chicago, IL, USA).

## 3. Results

In the first group, we treated the eye with the worst retinal sensitivity, including 78 eyes from 39 patients (16 females, 23 males) with a mean age of 56.46 years (range: 28–88 years) (Table 1). The average BCVA was 0.3 logMAR for distance vision (range: 2.0–0 logMAR) and 16.26 points (range: 12–6 points) for near vision, improving to 9.51 points with 1.64× magnification. The conditions causing visual impairment in these eyes were as follows: RP in twenty eyes, SMD in twelve, MMD associated with GON in ten, dry AMD in eight, GON in six, MMD in four, OA in four, DRP in four, CD in four, GAC in two, RCD in two, and OMD in two eyes (Table 1).

In this group, the 39 eyes (50%) treated with insulin (the worse-seeing eye) showed a significant increase in retinal sensitivity, from 10.09 ± 7.05 decibel (dB) to 10.75 ± 7.25 dB, representing a 6.54% improvement (*p* = 0.0067).

**Figure 2:**
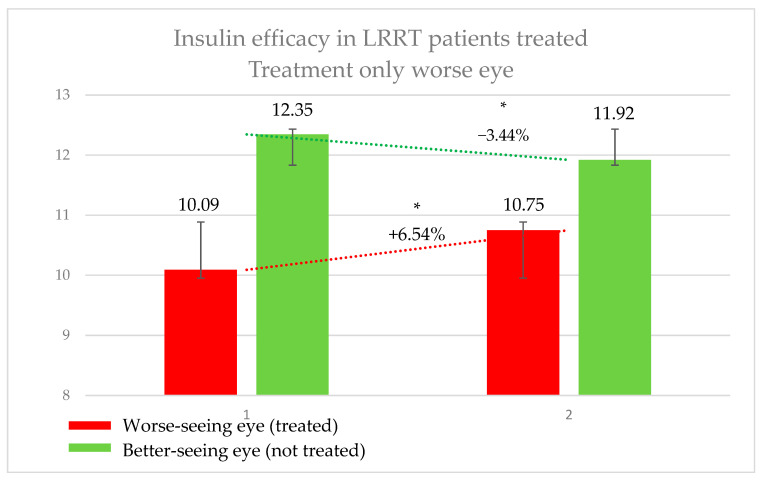
Efficacy of insulin treatment on the worse eye in patients undergoing the Limoli Retinal Restoration Technique (LRRT). Change in retinal sensitivity in decibels (dB) measured by microperimetry (MY) in the worse eye before and after insulin-enhanced injection. Error bars represent the standard deviation. Asterisk (*) indicates statistical significance at *p* < 0.05. The statistically significant improvement in sensitivity may support the potential therapeutic effect of insulin in neuroretinal degenerations.

Conversely, the 39 eyes (50%) that did not receive insulin (the better-seeing eye) exhibited a significant decrease in sensitivity, from 12.35 ± 6.79 dB to 11.92 ± 6.81 dB, corresponding to a 3.44% decline (*p* = 0.0448) (Figure 2). The difference in sensitivity change between treated and untreated eyes was 8.57%. The subjective score for the treated eyes was +0.50, compared to −0.13 for the untreated eyes. Similarly, the objective score was +0.51 for treated eyes and −0.10 for untreated eyes.

In the second group, which included 25 patients (13 females and 12 males) with a mean age of 56.81 years (range: 27–88 years), a total of 50 eyes were recruited (Table 1).

**Figure 3:**
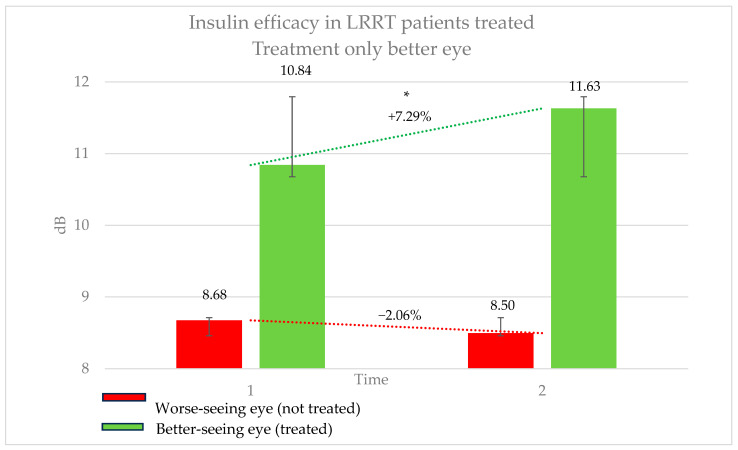
Efficacy of insulin treatment on the better eye in patients undergoing the Limoli Retinal Restoration Technique (LRRT). Change in retinal sensitivity in decibels (dB) measured by microperimetry (MY) in the better eye before and after insulin-enhanced injection. The results indicate a statistically significant improvement in sensitivity, highlighting the potential benefits of insulin treatment in neuroretinal degenerative conditions. Error bars represent the standard deviation. Asterisk (*) indicates statistical significance at *p* < 0.05. The statistically significant improvement in sensitivity may support the potential therapeutic effect of insulin in neuroretinal degenerations.

The average BCVA was 0.56 logMAR for distance vision (range: 2.0–0 logMAR) and 25.31 points for near vision (range: 12–6 points), improving to 10.71 points with 2.85× magnification. The conditions affecting these eyes were as follows: RP in ten eyes, SMD in ten, MMD associated with GON in eight, ON in six, dry AMD in four, GON in two, MMD in two, DRP in two, AS in two, RCD in two, and OMD in two eyes (Table 1). In this group, the treated eye (better-seeing eye) showed an increase in retinal sensitivity from 10.84 ± 6.66 dB to 11.63 ± 6.71 dB, corresponding to a 7.29% improvement (*p* = 0.05). Conversely, the untreated eye (worse-seeing eye) exhibited a decrease in sensitivity from 8.68 ± 7.20 dB to 8.50 ± 7.14 dB, representing a −2.06% change (*p* = 0.6771) (Figure 3). The difference in sensitivity change between treated and untreated eyes was 9.35%. The subjective score for the treated eye was +0.44, compared to −0.12 for the untreated eye. The objective score was +0.32 for the treated eye and −0.44 for the untreated eye.

In the third group, treated with a galenic insulin eye drop preparation, 26 patients (12 females and 14 males), with a mean age of 47.98 years (range: 23–82 years), and a total of 45 eyes were included (Table 1). The average BCVA was 0.51 logMAR for distance vision (range: 1.3–0 logMAR) and 16.80 points for near vision (range: 64–6 points), improving to 10.32 points with 1.43× magnification.

**Figure 4:**
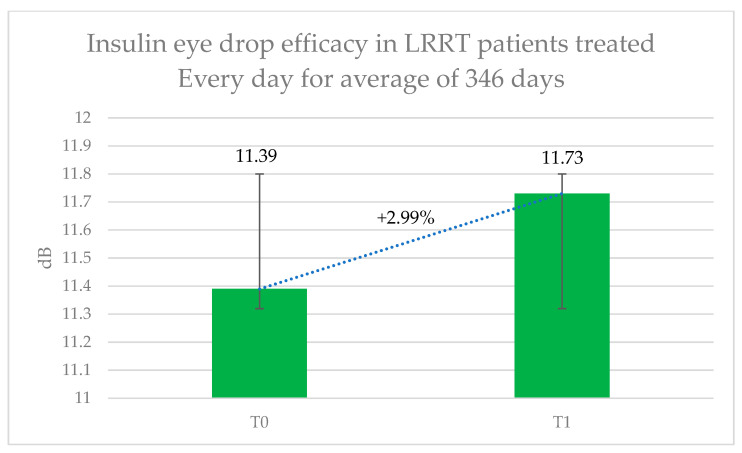
Therapeutic efficacy of long-term insulin eye drop treatment in patients previously undergoing the Limoli Retinal Restoration Technique (LRRT). Retinal sensitivity in decibels (dB) measured by microperimetry (MY) was assessed at baseline and after approximately one year of daily topical insulin therapy. Error bars represent the standard deviation. Although the results demonstrated modest improvement in retinal function, the increase was not statistically significant.

The visual impairments diagnosed in these eyes were as follows: RP in sixteen eyes, MMD associated with GON in eight, OA in seven, GON in three, SMD in four, AS in two, GAC in two, OMD in two, CD in two, MMD in two, and dry AMD in one eye (Table 1). In this group (Figure 4), retinal sensitivity improved from 11.39 ± 8.12 dB to 11.73 ± 8.28 dB, representing a 2.99% increase; however, this change was not statistically significant (*p* = 0.231). The subjective score for the treatment was +0.35, while the objective score for the treated eye was +0.08. Mean blood glucose levels were 126.44 mg/dL (range: 85–160) before treatment and 120.64 mg/dL (range: 76–152) after treatment.

## 4. Discussion

The aim of this study was to evaluate the therapeutic effects of insulin in patients with neuroretinal degenerations who had previously undergone LRRT, utilizing both insulin-enhanced platelet secretome injections and insulin-based eye drops. This patient population represents a suitable clinical target for such an investigation, as the scleral discontinuity created by the LRRT procedure facilitates effective pharmacological delivery—such as insulin—through the choroid to retinal tissues. Consequently, regular clinical and therapeutic monitoring over time was essential for assessing treatment outcomes.

This novel approach allows for the investigation of insulin’s potential therapeutic effects in pathological conditions such as neuroretinal degeneration. Our results suggest that insulin may serve as a viable treatment option to preserve visual function, likely through its metabolic support of neuroretinal cells, which are often compromised in various retinal diseases. In patients receiving insulin-based eye drops, retinal sensitivity was either stabilized or slightly improved, indicating a halt in functional decline due to disease progression (Figure 2 and Figure 3), in both better- and worse-seeing eyes, respectively. Importantly, no local or systemic adverse effects were reported in patients treated with insulin-enhanced secretome injections and insulin eye drops, supporting the safety profile of this therapeutic strategy.

The choice of a 5-unit dose of insulin lispro mixed with the secretome was based on clinical considerations and safety.

Preclinical studies cited in the literature utilized pure insulin applied topically to the cornea in animal models. The addition of hyaluronic acid aimed to enhance patient comfort and improve treatment adherence.

The initial formulation was administered to a diabetic patient as a safety precaution. After observing no adverse effects and confirming good patient compliance, subsequent batches were produced by a specialized compounding pharmacy in Milan, maintaining the same composition of 80% Humalog and 20% hyaluronic acid (4%). Insulin lispro is commercially available at a concentration of 100 units/mL, and daily insulin requirements vary widely among diabetic patients depending on individual factors such as diabetes type, body weight, diet, and physical activity [28,29]. Typical daily doses range from approximately 0.3 to 1 unit/kg, resulting in total daily doses of 25–80 units or more.

Given that ocular application does not require systemic glycemic control, a significantly lower dose was chosen—5 units in 0.05 mL—corresponding to approximately 20% of the lowest typical daily dose used in diabetes management. This small dose was added to 1.5 mL of platelet-derived mesenchymal secretome to enhance therapeutic effects without systemic impact creating a “potentiated secretome.” Insulin, an analog of insulin-like growth factor (IGF), binds to the same receptor and is not expected to produce adverse interactions. Conversely, its higher receptor affinity may result in stronger activation of intracellular pathways involved in neuroprotection and metabolic support [36,37].

Importantly, this dose has not been associated with local or systemic adverse effects in our patients, supporting its safety and tolerability. To date, no formal dose-finding trials have been conducted, and future studies are needed to further refine the optimal dosing for ocular applications.

Several studies have documented the beneficial effects of insulin—administered systemically or topically—on degenerating neuroretinal tissues and ocular surface disorders. It is well established that RGCs undergo dendritic retraction, synaptic loss, and neuronal death following axonal injury.

Agostinone et al. reported that administration of recombinant human insulin, either topically via eye drops or systemically prior to neuronal loss, promoted dendritic regeneration and synaptic reconnections. Insulin-mediated regeneration of excitatory postsynaptic sites on RGC dendrites enhanced neuronal survival and restored light-evoked retinal responses [30]. Their findings demonstrated that insulin-dependent activation of the mTORC1 complex is critical for maintaining dendritic arbor complexity, while mTORC2 supports dendritic process elongation, thereby restoring the dendritic field area. The authors concluded that mammalian central nervous system neurons possess the capacity for dendritic and synaptic regeneration following injury, proposing insulin and its analogs as promising therapeutic agents capable of inducing pro-regenerative responses in currently untreatable neurodegenerative diseases such as GL [30].

Zhang et al. observed that insulin and IGF-1 play pivotal roles in osteogenesis and angiogenesis, particularly during bone regeneration following trauma, tumor resection, congenital malformations, or infections [31]. They proposed that sustained insulin release via nanoparticles carrying insulin/IGF represents a promising strategy for enhancing bone regeneration. Various delivery systems, such as hydroxyapatite, gelatin, poly lactic-co-glycolic acid (PLGA), chitosan, and alginate, can be employed to optimize the timing and control of insulin release. Given the complexity and extended duration of bone regeneration, localized delivery of insulin may provide significant therapeutic benefits [31].

El Hajji et al. demonstrated that daily administration of recombinant human insulin eye drops stimulated dendritic and synaptic regeneration in RGCs within ocular hypertension models, a well-established risk factor for GL [32]. Their study identified the ribosomal protein p70S6 kinase (S6K) as a key mediator of insulin-induced dendritic regeneration. In vivo two-photon retinal imaging revealed that insulin restored light-evoked calcium (Ca^2+^) signaling in RGCs, enhanced neuronal survival, and improved retina-to-brain connectivity. These findings support insulin as a promising pro-regenerative therapy with potential clinical applications in GL management [32].

Additionally, some studies have evaluated the efficacy of topical insulin for ocular surface disorders [38,39,40,41,42,43,44]. Topical insulin has demonstrated effectiveness in promoting healing of persistent epithelial defects, including neurotrophic corneal ulcers. Formulated in artificial tears at concentrations ranging from 1 IU/mL to 100 IU/mL, complete clinical resolution was reported in all cases, with healing times as rapid as 2.5 days. In more severe cases, such as chemical injuries, healing extended beyond 60 days [38,39,40]. Based on the existing literature and our preliminary data, insulin may activate biochemical pathways in degenerated neuro-retino-choroidal tissues, although the precise underlying mechanisms remain to be fully elucidated.

The rationale behind this study is supported by preclinical evidence indicating that topical application of insulin to the cornea promotes optic nerve fiber regrowth, as well as by its affinity for the same receptor as insulin-like growth factor 1 (IGF-1).

IGF-1 plays a critical role in the development and maintenance of the central nervous system, including the retina, by supporting cell proliferation, differentiation, and survival, particularly of photoreceptors. Dysregulation of IGF-1 signaling has been implicated in several retinal diseases, such as diabetic retinopathy and age-related macular degeneration.

Insulin, by binding to the IGF-1 receptor, enhances glucose uptake into neuroretinal cells. In visually impaired patients, large scotomas often result not only from irreversible neuronal loss but also from the dysfunction of surviving neurons that are metabolically impaired. These “silent” neurons may be inactive due to metabolic suppression caused by chronic inflammation. By increasing glucose uptake and ATP production, insulin may help reactivate these underperforming neurons.

This is supported by the observed improvement in retinal sensitivity measured by microperimetry in the days following insulin treatment. Since insulin has a higher affinity for the receptor than IGF-1, we proposed that adding a small amount of insulin to the stem cell-derived secretome could enhance its neuroprotective and metabolic effects [45,46].

Visual acuity measured by logMAR alone is often insufficient, as it assesses only central foveal function and may fail to detect extensive peripheral damage, as seen in conditions such as retinitis pigmentosa. Near visual acuity provides additional information by reflecting the patient’s ability to read groups of words, which requires a larger intact retinal area. However, retinal sensitivity measured by microperimetry remains the most reliable method, as it evaluates function across multiple retinal regions with high precision and repeatability, facilitated by the device’s eye-tracking capabilities.

Insulin may reduce oxidative stress indirectly by enhancing cellular glucose uptake and metabolic activity, thereby supporting endogenous antioxidant systems. Additionally, insulin signaling plays a neurotrophic role in the retina, with studies demonstrating that the loss of insulin receptors can lead to photoreceptor degeneration. Furthermore, evidence suggests that insulin produced by the retinal pigment epithelium supports photoreceptor energy demands, contributing to mitochondrial function and cell survival [47,48].

Thus, insulin plays a key neurotrophic role in the retina by promoting neuronal survival and modulating apoptosis. In diabetic conditions, impaired retinal insulin signaling contributes to neurodegeneration and may lead to progressive vision loss. These findings support the rationale for investigating insulin as a neuroprotective agent in retinal diseases [49].

Insulin is crucial for mitochondrial protection and function. It regulates mitochondrial proteostasis, enhances oxidative phosphorylation, and promotes mitochondrial biogenesis, thereby increasing ATP production. Insulin resistance, as observed in type 2 diabetes, impairs mitochondrial function, underscoring insulin’s essential role in maintaining mitochondrial health [50,51,52].

The limitations of our preliminary study include the relatively small sample size and the heterogeneity of the posterior segment pathologies included. Additionally, the combined administration of insulin via platelet secretome injections and topical eye drops introduces a confounding factor that requires separate evaluation in future studies. Furthermore, the one-year follow-up period may be insufficient to fully capture the potential long-term benefits, especially given the prolonged timelines generally required for neuroregeneration in damaged retinal tissues. Finally, it remains unclear whether the insulin-containing eye drops used in patients with neuroretinal diseases effectively reach and are activated by the cells of the posterior segment.

While these factors cannot be entirely ruled out, insulin injection was a one-time intervention for each patient, and microperimetry was performed within a few days to objectively assess immediate functional changes.

This study is based on clinical cases treated for low-vision rehabilitation at the Low Vision Study Center in Milan. Although no separate control group was included, the untreated eye in groups 1 and 2 served as an internal control for comparison with the insulin-treated eye. The observed increase in retinal sensitivity in treated eyes compared to untreated eyes suggests a potential therapeutic effect of insulin in neuroretinal diseases characterized by impaired sensory function.

However, the small sample size limits the generalizability of these findings and highlights the need for further, large-scale research. Therefore, this work should be considered a pilot study.

Numerous researchers have reported the healing of corneal defects in human subjects following insulin instillation, as well as potential benefits in experimental models of neuroretinal damage. However, these findings remain preliminary, supported by limited studies and largely speculative hypotheses due to the absence of long-term, well-established histopathological investigations [30,31,32,40,41,42,43,44]. Our preliminary study, inspired by these results, has demonstrated stabilization in some patients and modest clinical improvements in others affected by various neurodegenerative retinal diseases. These encouraging findings warrant further investigation to confirm their validity and to elucidate the underlying biochemical mechanisms by which insulin may support cellular stabilization and visual function improvement in damaged neuroretinal tissues.

## 5. Conclusions

In summary, although preliminary, our findings suggest a potential stabilizing effect of insulin—likely mediated through its metabolic influence on neuroretinal cells—consistent with a limited but growing body of evidence supporting its use in both anterior and posterior segment ocular diseases.

The primary outcome of this study is that the administration of 5 units of insulin in eye drops resulted in a statistically significant improvement in retinal sensitivity, with an average increase of approximately 6%. While these findings are promising, they represent preliminary data from a pilot study.

Further research involving larger cohorts, longer follow-up periods, and controlled study designs across distinct neuroretinal pathologies and disease stages is essential to better elucidate the mechanistic relationship between insulin signaling and neuroretinal cell physiology.

Therefore, studies are warranted to confirm the therapeutic potential of insulin and clarify its underlying mechanisms of action.

## Figures and Tables

**Table 1:** Demographic and clinical characteristics of patients by group (64 subjects).

Type of Disease	Group 1 (*n* = 39)	Group 2 (*n* = 25)	Group 3 (*n* = 26)	
Glaucomatous optic neuropathy (GON)	3 (7.69%)	1 (4.0%)	2 (7.69%)	
Angioid streaks	0 (0.0%)	1 (4.0%)	1 (3.8%)	
Myopic macular degeneration	2 (5.13%)	2 (8.0%)	1 (3.8%)	
Myopic macular degeneration + GON	5 (12.82%)	4 (16.0%)	4 (15.4%)	
Rod–cone dystrophy	1 (2.56%)	1 (4.0%)	0 (0.0%)	
Stargardt’s macular degeneration	6 (15.38%)	5 (20.0%)	2 (7.7%)	
Dry age-related macular degeneration	4 (10.26%)	2 (8.0%)	1 (3.8%)	
Optic atrophy	2 (5.13%)	3 (12.0%)	4 (15.4%)	
Retinitis pigmentosa	10 (25.64%)	5 (20.0%)	8 (30.8%)	
Occult macular dystrophy	1 (2.56%)	1 (4.0%)	1 (3.8%)	
Diabetic retinopathy	2 (5.13%)	1 (4.0%)	0 (0.0%)	
Gyrate atrophy of the choroid	1 (2.56%)	0 (0.0%)	1 (3.8%)	
Cone dystrophy	2 (5.13%)	0 (0.0%)	1 (3.8%)	
				*p*
Age (in years) mean (±SD)	56.46 (±17.4)	56.81 (±18.5)	47.98 (±18.2)	0.694
Female, n (%)	16 (41.0)	13 (52.0)	12 (46.2)	0.060
Male, n (%)	23 (59.0)	12 (48.0)	14 (53.8)	0.060
BCVA, logMar mean (±SD) t0	0.49 (0.33)	0.56 (0.99)	0.51 (0.34)	0.290
BCVA, logMar mean (±SD) t1	0.49 (0.32)	0.56 (0.99)	0.54 (0.41)	0.312
Sensitivity (dB) t0 mean (±SD)	11.22 (6.98)	9.75 (6.95)	11.39 (8.12)	0.526
Sensitivity (dB) t1 mean (±SD)	11.33 (7.01)	10.06 (7.05)	11.73 (8.27)	0.713

BCVA: best-corrected visual acuity; logMAR: logarithm of the minimum angle of resolution; dB: decibel. Statistical tests: one-Way ANOVA for independent measures and Fisher’s exact test. Significance threshold *p* < 0.05.

## Data Availability

Data supporting reported results can be obtained from Limoli P.G.

## Abbreviations

The following abbreviations are used in this manuscript:

ADSCs	Adipose-derived stem cells
AKT = PKB	Protein kinase B
AMD	Age-related macular degeneration
AS	Angioid streaks
ASCs	Adipose stromal cells
ATP	Adenosine triphosphate
BCVA	Best-corrected visual acuity
Ca^2+^	Calcium
CD	Cone dystrophy
dB	Decibel
DNA	Deoxyribonucleic acid
DRP	Diabetic retinopathy
EMEA	European Medicines Evaluation Agency
ERK	Extracellular signal-regulated kinase
FDA	Food and Drug Administration
GAC	Gyrate atrophy of the choroid
GFs	Growth factors
GL	Glaucoma
GLUT1/3	Facilitative glucose transporters
GON	Glaucomatous optic neuropathy
IGFs	Insulin-like growth factors
IRB	Institutional Review Board
LogMAR	Logarithm of the minimum angle of resolution
LRRT	Limoli Retinal Restoration Technique
MEK	Mitogen-activated extracellular signal-regulated kinase
MMD	Myopic macular degeneration
mRNA	Messenger ribonucleic acid
mTORC	Serine/threonine kinase complex
MY	Microperimetry
OA	Optic atrophy
OMD	Occult macular dystrophy
p110–p85	Heterodimer, key component of phosphoinositide 3-kinases (PI3K)
PI3K/AKT	Phosphatidylinositol 3-Kinase/Protein Kinase B intracellular signaling pathway
PIP2	Phosphatidylinositol 4,5-bisphosphate
PLGA	Poly lactic-co-glycolic acid
PRP	Platelet rich plasma
RCD	Rod–cone dystrophy
RGCs	Retinal ganglion cells
RP	Retinitis pigmentosa
S6K	Ribosomal protein p70S6 kinase
SD	Standard deviation
SMD	Stargardt’s macular degeneration
